# *Ent*-Peniciherqueinone Suppresses Acetaldehyde-Induced Cytotoxicity and Oxidative Stress by Inducing ALDH and Suppressing MAPK Signaling

**DOI:** 10.3390/pharmaceutics12121229

**Published:** 2020-12-18

**Authors:** Taehoon Oh, Mincheol Kwon, Jae Sik Yu, Mina Jang, Gun-Hee Kim, Ki Hyun Kim, Sung-Kyun Ko, Jong Seog Ahn

**Affiliations:** 1 Natural Medicine Research Center, Korea Research Institute of Bioscience and Biotechnology (KRIBB), Cheongju 28116, Korea; dhxogns10@kribb.re.kr; 2 College of Pharmacy, Chungbuk National University, Cheongju, Chungbuk 28160, Korea; 3 Anticancer Agent Research Center, Korea Research Institute of Bioscience and Biotechnology (KRIBB), Cheongju 28116, Korea; mckwon@kribb.re.kr (M.K.); jangmina@kribb.re.kr (M.J.); kimgh@kribb.re.kr (G.-H.K.); 4 Department of Biomolecular Science, KRIBB School of Bioscience, Korea University of Science and Technology (UST), Daejeon 34141, Korea; 5 School of Pharmacy, Sungkyunkwan University, Suwon 16419, Korea; jsyu@bu.edu

**Keywords:** *ent*-peniciherqueinone, acetaldehyde, oxidative stress, anti-oxidation

## Abstract

Studies on ethanol-induced stress and acetaldehyde toxicity are actively being conducted, owing to an increase in alcohol consumption in modern society. In this study, *ent*-peniciherqueinone (EPQ) isolated from a Hawaiian volcanic soil-associated fungus *Penicillium herquei* FT729 was found to reduce the acetaldehyde-induced cytotoxicity and oxidative stress in PC12 cells. EPQ increased cell viability in the presence of acetaldehyde-induced cytotoxicity in PC12 cells. In addition, EPQ reduced cellular reactive oxygen species (ROS) levels and restored acetaldehyde-mediated disruption of mitochondrial membrane potential. Western blot analyses revealed that EPQ treatment increased protein levels of ROS-scavenging heme oxygenase-1 and superoxide dismutase, as well as the levels of aldehyde dehydrogenase (ALDH) 1, ALDH2, and ALDH3, under acetaldehyde-induced cellular stress. Finally, EPQ reduced acetaldehyde-induced phosphorylation of p38 and c-Jun N-terminal kinase, which are associated with ROS-induced oxidative stress. Therefore, our results demonstrated that EPQ prevents cellular oxidative stress caused by acetaldehyde and functions as a potent agent to suppress hangover symptoms and alcohol-related stress.

## 1. Introduction

Alcohol consumption has increased globally, and it causes a variety of physical side effects and social problems. Ethanol enters the bloodstream via absorption in the stomach and small intestine, after which it is distributed across all body organs [1,2]. Until this phase, alcohol dehydrogenase (ADH) in the liver cells rapidly converts alcohol to acetaldehyde [3,4]. However, until acetaldehyde is metabolized by aldehyde dehydrogenases (ALDHs) and converted into water and acetic acid [3,5,6], it circulates in the bloodstream and strongly induces cytotoxicity and oxidative stress in organs such as the liver and brain [2,7,8,9,10]. Moreover, acute acetaldehyde exposure causes nausea, vomiting, dizziness, and muscle pain and chronic acetaldehyde exposure cause severe liver and brain cell damage [7,8,11]. ALDH is the main enzyme involved in the detoxification and metabolism of acetaldehyde to acetate [11,12]. ALDH is present in various types of tissues and efficiently metabolizes acetaldehyde. Therefore, preserving the levels of ALDH is an important mechanism for protecting tissues from the effects of acetaldehyde.

Ethanol and acetaldehyde induce the activation of the mitogen-activated protein kinase (MAPK) pathway mediated through reactive oxygen species (ROS), in the liver and neuronal cells [13,14,15]. The MAPK cascade is a central signaling pathway that responds directly to a variety of cellular stimuli in the body [16,17,18,19]. Three well-characterized subfamilies of MAPK are the extracellular signal-regulated kinase (ERK), p38, and c-Jun N-terminal kinase (JNK) [18]. The MAPK family plays key roles in cellular processes such as senescence, differentiation, proliferation, development, inflammatory response, apoptosis, and oxidative stress [18,19]. The activation of the MAPK pathway induced by acetaldehyde results in cytotoxicity and tissue collapse, leading to alcoholic dementia and hangovers [20,21]. Therefore, compounds that inhibit ethanol or acetaldehyde-mediated MAPK signal activation may be potential candidates for neuronal cell protection and hangover relief.

As part of our continuing projects to discover bioactive natural products from diverse natural sources [22,23,24,25,26], a soil-associated fungal strain *Penicillium herquei* FT729 was isolated from a soil sample collected at Hualālai, an active volcano on Big Island, Hawaii. *P. herquei* produces distinct fungal phenalenones, which are a unique class of aromatic ketones comprised of a three-ring system, and the phenalenone derivatives have been reported to exhibit a variety of biological effects including anticancer and antimicrobial activities [27]. LC/MS-guided chemical analysis of a MeOH extract of *P. herquei* FT729 resulted in the isolation of a phenalenone derivative through successive column chromatography with preparative and semi-preparative HPLC. The isolated compound was structurally elucidated as *ent*-peniciherqueinone (EPQ), based on the NMR spectroscopic data [28] and LC/MS analysis. EPQ is a bioactive compound with anti-inflammatory, anti-angiogenic, and adipogenesis-inducing properties [28]. In this study, we investigated whether EPQ can confer protection against acetaldehyde-induced cytotoxicity and oxidative damage in PC12 cells. To determine the mechanisms underlying the inhibition of acetaldehyde activity using EPQ, we evaluated the effects of EPQ on the expression of ROS-scavenging genes and aldehyde dehydrogenase genes, in addition to its effects on MAPK phosphorylation. 

## 2. Materials and Methods

### 2.1. Purification and Identification of Ent-Peniciherqueinone (EPQ)

**Figure 1:**
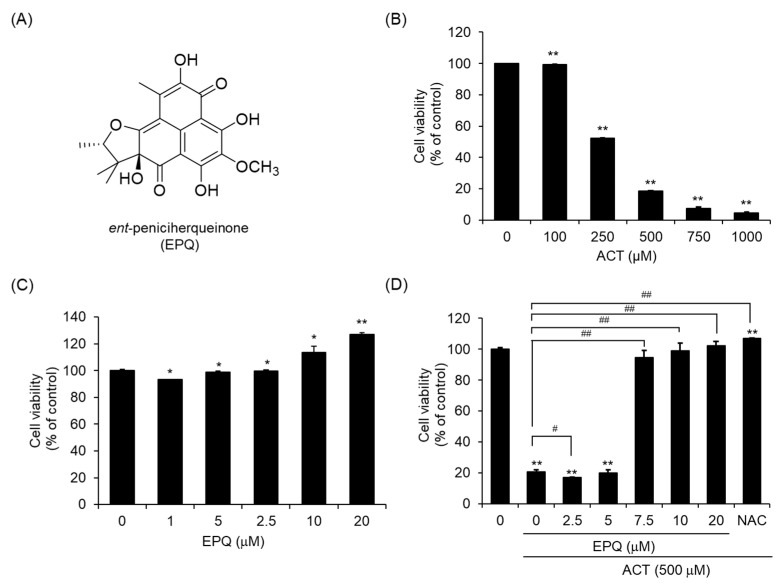
Effects of EPQ on acetaldehyde-induced cytotoxicity in PC12 cells. (**A**) Chemical structure of EPQ. (**B**) Cells were treated with the indicated concentrations of acetaldehyde for 24 h. All values are expressed as mean ± standard error of the mean (SEM) (*n* = 3; ** *p* < 0.01 compared to the DMSO control group). (**C**) Cells were seeded on a 96-well plate and treated with various concentrations of EPQ for 24 h. All values are expressed as mean ± SEM (*n* = 3; * *p* < 0.05 compared to the DMSO control group). (**D**) Cells were treated with various concentrations of EPQ in the presence or absence of acetaldehyde (500 μM) for 24 h. NAC (5 mM) was used as a positive control. All values are expressed as mean ± SEM (*n* = 3; ** *p* < 0.01 compared to the DMSO control group and # *p* < 0.05, ## *p* < 0.01 compared to the group treated only with ACT). ACT, acetaldehyde; EPQ, *ent*-peniciherqueinone; DMSO, dimethyl sulfoxide; NAC, *N*-acetyl-l-cysteine.

The fungal strain *P. herquei* FT729 was isolated and identified as described in detail in our previous study [29]. The cultivation was performed using the method described in the study [30]. LC/MS analysis of mycelia and broth liquid solutions of *P. herquei* FT729 revealed that both showed no significant difference in the chemical profile. They were combined and extracted with 80% MeOH/H_2_O, then concentrated using a rotavapor to obtain 12.5 g of crude extract. The crude extract was suspended in distilled water and applied to solvent-partitioning with ethyl acetate (EtOAc) and *n*-butanol (BuOH) to obtain two main fractions, EtOAc-soluble (3.8 g) and BuOH-soluble (1.0 g) fractions. Both fractions were examined under LC/MS analysis with a combination of our in-house UV library, and the EtOAc-soluble fraction was considered a significant target for isolation. The EtOAc-soluble fraction (3.8 g) was fractionated using silica gel column chromatography [eluted with CH_2_Cl_2_/MeOH (100:1 → 1:1 → 100% MeOH) of the gradient solvent system] to provide seven fractions (A1–A7). Fraction A4 (322.3 mg) was subjected to Sephadex LH-20 [eluted with CH_2_Cl_2_/MeOH (1:1) of the isocratic solvent system] to provide four subfractions (A41-A44). The subfraction A42 (100.7 mg) was further fractionated with preparative reversed-phase HPLC with MeOH/H_2_O (7:10 → 100% MeOH) of the gradient solvent system to produce six subfractions (A421-A426). Finally, subfraction A423 (56.1 mg) was further purified by semi-preparative HPLC (75% MeOH) to furnish *ent*-peniciherquinone (*t_R_* 35.5 min, 0.5 mg). The structure of *ent*-peniciherquinone (EPQ, Figure 1A) was identified by comparing their physical and NMR spectroscopic data with those reported in the literature [28] and determined using LC/MS analysis.

### 2.2. Cell Culture

PC12 (Pheochromocytoma of rat adrenal medulla) cells were purchased from the American Type Culture Collection (ATCC). The cells were routinely cultured in Dulbecco’s modified Eagle’s medium (DMEM; Welgene, Gyeongsan, Korea, LM 001-05) containing 10% fetal bovine serum (FBS; Welgene, Gyeongsan, Korea, S001-07), 100 units penicillin, and 100 μg/mL streptomycin (Gibco, Grand Island, NY, USA, 15140-122) at 37 °C in a humidified atmosphere containing 5% CO_2_.

### 2.3. Antibodies and Reagents

The primary antibodies were purchased from these resources: Heme oxygenase-1 (HO-1; Santa Cruz, CA, USA, sc-136960), superoxidase dismutase 2 (SOD2; Santa Cruz, CA, USA, sc-133134), aldehyde dehydrogenase (ALDH) 1A1 (Santa Cruz, CA, USA, sc-374076), ALDH2A1 (Santa Cruz, CA, USA, sc-100496), ALDH3A1 (Santa Cruz, CA, USA, sc-376089), and GAPDH (Santa Cruz, CA, USA, sc-47724). The secondary antibodies were horseradish peroxidase-conjugated anti-mouse IgG (Cell Signaling Technology, 7076) and anti-rabbit IgG (Cell Signaling Technology, 7074). An Annexin V-FITC Apoptosis Detection kit was purchased from eBioscience (San Diego, CA, USA). Dimethylsulfoxide (DMSO, # 41639), 1,1-Diphenyl-2-picryl-hydrazyl (DPPH), and ascorbic acid were purchased from Sigma-Aldrich (St. Louis, MO, USA). GSH-Glo assay kit (Promega, Madison, WI, USA, # V6911) and ROS-Glo™ H_2_O_2_ assay kit (Promega, Madison, WI, USA, #G8820) were purchased from Promega (Madison, WI, USA, V6911).

### 2.4. Cell Viability Assay and Anti-Acetaldehyde Activity Assay

Cell viability and anti-acetaldehyde activity were determined using the EZ-Cytox colorimetric assay (Daeil Lab service, 0793, Seoul, Korea), according to a previous report [30]. For the cytotoxicity and anti-acetaldehyde activity assays, PC12 cells were cultured in 96-well plates (1.5 × 10^4^ cells/well) for 12 h. Cells were treated with DMSO (0.5%, *v*/*v*), EPQ (0.5% of DMSO, *v*/*v*), acetaldehyde (0.5% *v*/*v*), or co-treated with EPQ and acetaldehyde (500 μM) at the indicated concentrations for 24 h. After treatment, cells were washed with phosphate-buffered saline (PBS) and replaced with EZ-Cytox solution, and incubated for 1 h. Live cells were read in a microplate reader (Molecular Devices, Spectra Max 190, San Jose, CA, USA) at 450 nm. Cell viability was normalized using the DMSO control.

### 2.5. Nuclear Staining with Hoechst 33342 and Propidium Iodide (PI)

PC12 cells were seeded to 48-well plates (2 × 10^4^ cells/well) and treated with DMSO (0.5%, *v*/*v*) or indicated dose of EPQ (0.5% of DMSO, *v*/*v*) in the presence or absence of acetaldehyde (500 μM) for 24 h. After treatment, cells were washed with PBS and stained with Hoechst 33342 (25 μg/mL, Invitrogen, Carlsbad, CA, USA, H21492) and PI (5 μg/mL, Invitrogen, Carlsbad, CA, USA, P21493) for 30 min at 37 °C. Fluorescent images were captured under the fluorescence cell imaging system (Thermo Fisher Scientific, EVOS FL, Waltham, MA, USA).

### 2.6. Annexin V/PI Staining

PC12 cells were seeded to 6-well plates (3 × 10^5^ cells/well) and treated with DMSO (0.5%, *v*/*v*) or EPQ (0.5% in DMSO, *v*/*v*) in the presence or absence of acetaldehyde (500 μM) for 24 h. After treatment, cells were washed with PBS and suspended with trypsin-EDTA (0.5 mL, 0.05% trypsin, 0.02% EDTA, Sigma-Aldrich, Saint Louis, MO, USA) for 5 min at 37 °C and collected. The cells were re-suspended in a binding buffer (500 μL, 10 mM HEPES/NaOH, pH 7.5 containing 1.4 M NaCl and 2.5 mM CaCl_2_) and stained with annexin V-FITC (0.5 μg/mL) and PI (2 μg/mL) for 15 min at room temperature. The stained cells were determined using the BD FACSCalibur™ flow cytometer (BD Biosciences, Franklin Lake, NJ, USA) and the data were analyzed using FlowJo v10 (BD Biosciences, Franklin Lake, NJ, USA).

### 2.7. DPPH Assay

The 1,1-diphenyl-2-picryl-hydrazyl (DPPH) assay was performed to determine whether EPQ suppressed acetaldehyde-induced ROS generation according to the manufacturer’s instructions [30]. DPPH (150 μM) stock solution was prepared in methanol. Various concentrations of EPQ (0.5% of DMSO, *v*/*v*) or ascorbic acid (AA, 1 mM) (50 µL) were added to a 96-well plate containing 150 µL of DPPH stock solution (Total 200 µL) and incubated for 60 min. The samples were analyzed using a microplate reader (Molecular Devices, SpectraMax 190) at 517 nm. The DPPH free radical-scavenging activity was calculated according to equation 1.

(1) $$DPPH\;\mathrm{radical}\;scavaging\;\mathrm{activity}\;\left(\%\;inhibition\right)=\frac{A0-A1}{A0}\times 100$$

where, *A*0 is the absorbance of the DPPH control reaction, and A1 is the absorbance in the candidate sample.

### 2.8. In Vitro GSH-Glo Assay

Reduced and oxidized GSH levels were determined using the GSH-Glo assay kits according to the manufacturer’s instructions [31]. PC12 cells (1 × 10^4^ cells/mL) were seeded to 96-well plates for 12 h. The cells were treated with 0–20 μM of EPQ (0.5% of DMSO, *v*/*v*) in the presence or absence of acetaldehyde (500 μM) for 24 h. The treated cells were washed with PBS and incubated with 2X GSH-Glo™ Reagent (50 µL) for 30 min. The luminescence of the samples was measured using a luminescence plate reader (VictorTM X2, PerkinElmer, Waltham, MA, USA).

### 2.9. Cellular H_2_O_2_ Generation Assay

The cellular production of hydrogen peroxide (H_2_O_2_) was measured in PC12 cells using the ROS-Glo™ H_2_O_2_ assay kit according to the manufacturer’s instructions [32]. Briefly, PC12 cells (1 × 10^4^ cells/mL) were seeded to 96-well plates and incubated overnight. The cells were treated with various concentrations of EPQ (0.5% of DMSO, *v*/*v*) in the presence or absence of acetaldehyde (500 μM) for 24 h, and the ROS-Glo H_2_O_2_ detection substrate was then added to the wells for 20 min. The luminescence of the samples was measured using a luminescence plate reader (VictorTM X2, PerkinElmer, Waltham, MA, USA).

### 2.10. Measurement of Intracellular Oxidative Stress

The fluorescent probe 2′,7′-dichlorodihydrofluorescein diacetate (DCFH-DA, Invitrogen, Carlsbad, CA, USA, #D399) was used to determine the changes in the intracellular generation of ROS according to the manufacturer’s instructions [33]. PC12 cells were seeded to a 48-well plate (5 × 10^5^ cells/well) and treated with DMSO (0.5% *v*/*v*) or EPQ (0.5% of DMSO, *v*/*v*) in the presence or absence of acetaldehyde (500 μM). After 24 h of incubation, the treated cells were washed with PBS and suspended with DCFH-DA (5 µM) for 30 min. The treated cells were performed using the BD FACSCalibur ™ Flow cytometry (BD Biosciences, USA) and the analysis data were processed using FlowJo v10 (BD Biosciences, Franklin Lake, NJ, USA).

### 2.11. Detection of Mitochondrial Membrane Potential (*Δ*Ψm)

JC-1 dye was used to measure the alterations in mitochondrial membrane potential (ΔΨm), according to our previous report [34]. PC12 cells were incubated with JC-1 (2.5 µg/mL, Anaspec, Fremont, CA, USA, # AS-88060,) for 15 min, and the treated cells were washed with PBS twice. The treated cells were performed using the BD FACSCalibur™ Flow cytometry (BD Biosciences, Franklin Lake, NJ, USA) and the flow cytometry data were processed using FlowJo v10 (BD Biosciences, Franklin Lake, NJ, USA).

### 2.12. Western Blot Analysis

PC12 cells were lysed with cold M-PER buffer (Thermo Fisher Scientific, Rockford, IL USA,#78501,) containing both protease inhibitor cocktail (Roche Diagnostics GmbH, 5056489001, Mannheim, Germany), and phosphatase inhibitor cocktail 3 (Sigma-Aldrich, St. Louis, MO, P0044) and then centrifuged at 13,500 rpm for 5 min at 4 °C. The samples were separated by 8–12% SDS-PAGE gel and electro-transferred onto nitrocellulose membranes (0.2 μm, Bio-Rad, 162-0112, USA). After blocking with 5% non-fat dry milk in tris-buffered saline containing 0.1% TWEEN 20 (TBST) buffer for 1 h, the membranes were incubated with the indicated primary antibodies at 4 °C for 12 h and then incubated with horseradish peroxidase-conjugated anti-mouse (1:5000) or anti-rabbit IgG (1:5000) for 1 h. The protein bands were visualized using the SuperSignal West Pico chemiluminescent substrate or SuperSignal West Femto maximum sensitivity substrate (Thermo Fisher Scientific, Rockford, IL, USA, #34080/#34095).

### 2.13. Statistics

Data are presented as mean ± standard error of the mean (SEM) of at least three independent experiments (*n* = 3). Statistical analyses were performed using GraphPad Prism (GraphPad Software, 8.4.3, San Diego, CA, USA). The differences were considered statistically significant at * *p* < 0.05, ** *p* < 0.01, when compared to the DMSO control group and at ^#^ *p* < 0.05, ^##^ *p* < 0.01 when compared to the group treated only with ACT.

## 3. Results

### 3.1. EPQ Effectively Suppresses Acetaldehyde-Induced Cytotoxicity in PC12 Cells

To investigate whether EPQ can effectively suppress acetaldehyde-induced cytotoxicity, we performed cell viability assays using PC12 cells. The cells were cultured and treated with various doses of acetaldehyde for 24 h and cell viability was determined using the EZ-Cytox colorimetric assay. As shown in Figure 1B, acetaldehyde exhibited strong cytotoxicity at an approximate IC_50_ value of 310.60 ± 0.08 μM. Based on this result, a concentration of 500 μM, with observed cytotoxicity of 80% or higher, was then selected as the cytotoxic concentration for acetaldehyde. To test whether EPQ affects cell viability, the PC12 cells were treated with 1, 2.5, 5, 10, and 20 μM EPQ for 24 h. EPQ did not result in any significant change in cell viability at concentrations of up to 20 μM (Figure 1C). To test the protective effect of EPQ against acetaldehyde-induced cytotoxicity, cell viability was analyzed using the EZ-Cytox colorimetric assay. The cells were co-treated with 500 μM acetaldehyde and 0–20 μM EPQ for 24 h. The cytotoxicity induced by acetaldehyde was significantly reduced by EPQ, at a concentration of 7.5 μM (Figure 1D).

**Figure 2:**
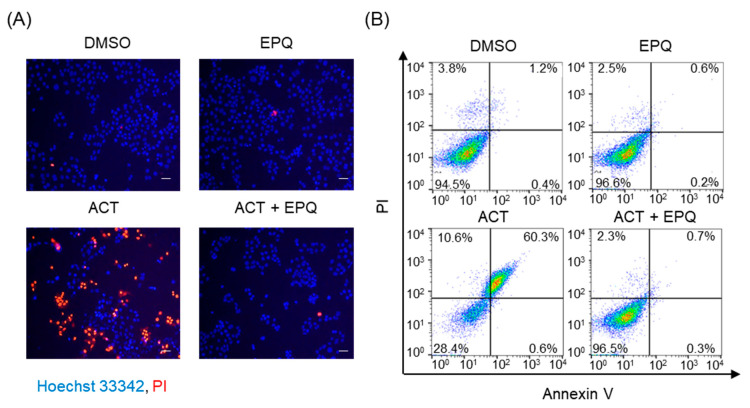
Inhibitory effects of EPQ on acetaldehyde-induced cytotoxicity in PC12 cells. (**A**) The effects of EPQ on the nuclear morphological changes of PC12 cells were observed by double staining with Hoechst 33342 (Blue) and propidium iodide (PI, Red). Cells were treated with DMSO (0.5%, *v*/*v*) or EPQ (20 μM) in the presence or absence of acetaldehyde (500 μM) and observed under a fluorescence cell imaging system. Scale bar = 50 μm. (**B**) Cells were treated with DMSO (0.5%, *v*/*v*) or EPQ (20 μM) in the presence or absence of acetaldehyde (500 μM) for 24 h. Apoptotic cells were stained with annexin V and PI stains and analyzed using flow cytometry. ACT, acetaldehyde; EPQ, *ent*-peniciherqueinone; DMSO, dimethyl sulfoxide; PI, propidium iodide.

To further examine the effects of EPQ on acetaldehyde-induced cell injury, we observed the morphological changes in PC12 cell nuclei. The cells treated with EPQ in the presence and absence of acetaldehyde were double-stained with Hoechst 33342 and PI for live-cell imaging. As shown in Figure 2A, no abnormal cells were observed in both DMSO and EPQ treatment groups; however, the cells treated with acetaldehyde alone exhibited typical necrosis-like morphological changes. However, co-treatment of cells with EPQ and acetaldehyde suppressed the cell death morphology. In addition, we confirmed that EPQ reduced acetaldehyde-induced cytotoxicity using flow cytometry analysis of annexin V/PI stained cells. Our results showed that after treatment with acetaldehyde alone, the percentage of early apoptotic and late apoptotic cells (70.9%) was higher than the control (5.0%). However, co-treatment of cells with EPQ and acetaldehyde reduced the percentage of early apoptotic and late apoptotic cells (3.0%) (Figure 2B). These results suggested that EPQ markedly suppressed the acetaldehyde-induced cytotoxicity in PC12 cells.

### 3.2. Protective Effects of EPQ against Acetaldehyde-Induced Oxidative Stress

**Figure 3:**
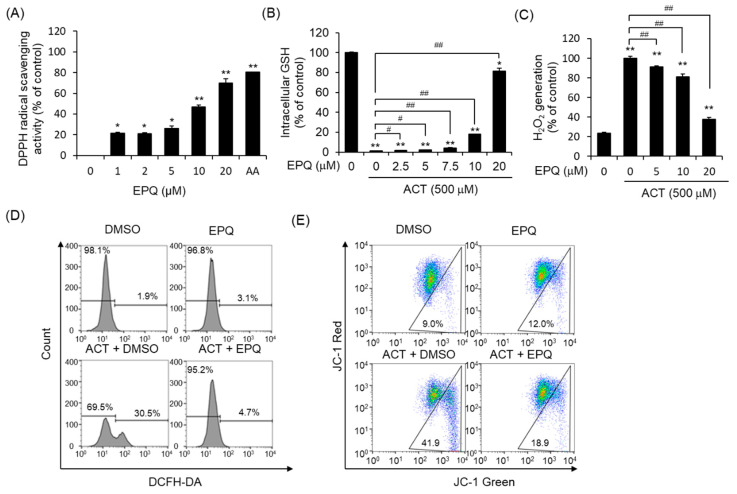
Inhibitory effects of EPQ on acetaldehyde-induced oxidative stress in PC12 cells. (**A**) The radical-scavenging activity of EPQ was measured using the DPPH assay. Cells were seeded in a 96-well plate and treated with various concentrations of EPQ for 24 h. AA (1 mM) was used as a positive control. All values are expressed as mean ± SEM (*n* = 3; * *p* < 0.05, ** *p* < 0.01 compared to the DMSO control group). (**B**) Intracellular GSH activity was measured using the GSH-Glo assay. Cells were treated with various concentrations of EPQ in the presence and absence of acetaldehyde (500 μM) for 24 h. All values are expressed as mean ± standard error (*n* = 3; ** *p* < 0.01 compared to the DMSO control group and ^#^ *p* < 0.05 compared to the group treated only with ACT). (**C**) H_2_O_2_ production was measured using the ROS-Glo H_2_O_2_ assay. Cells were treated with various concentrations of EPQ in the presence and absence of acetaldehyde (500 μM) for 24 h. All values are expressed as mean ± SEM (*n* = 3; ** *p* < 0.01 compared to the DMSO control group and ^##^ *p* < 0.01 compared to the group treated only with ACT). (**D**) The intensity of fluorescence of cells stained with DCFH-DA was analyzed using flow cytometry. Cells were treated with DMSO (0.5%, *v*/*v*) or EPQ (20 μM) in the presence and absence of acetaldehyde (500 μM) for 24 h. (**E**) Mitochondrial membrane potentials were detected using JC-1 staining and analyzed using flow cytometry. Cells were treated with DMSO (0.5%, *v*/*v*) or EPQ (20 μM) in the presence and absence of acetaldehyde (500 μM) for 24 h. ACT, acetaldehyde; EPQ, *ent*-peniciherqueinone; DMSO, dimethyl sulfoxide; AA, ascorbic acid; GSH, glutathione.

To determine the antioxidant potential of EPQ, we evaluated the effect of EPQ on free radical-scavenging activity using the 1,1-diphenyl-2-picryl-hydrazyl (DPPH) assay. EPQ treatment resulted in a significant increase in the DPPH radical-scavenging activity of cells at an approximate EC_50_ of 12.17 μM ± 2.38 µM (Figure 3A). Next, to determine whether EPQ inhibits acetaldehyde-induced ROS generation, we evaluated using the GSH-Glo and ROS-Glo H_2_O_2_ assay kits. Tripeptide Glutathione (GSH) is essential for the detoxification of ROS and maintaining the redox status of the cell. Acetaldehyde treatment significantly decreased GSH levels compared to that of the control in PC12 cells (Figure 3B). However, EPQ treatment rescued the levels of GSH decreased by acetaldehyde. Hydrogen peroxide (H_2_O_2_ generated as a by-product of metabolism, is the most abundant ROS species within cells, and plays an essential role in cellular oxidative stress. Therefore, the ROS-Glo H_2_O_2_ assay was performed to determine whether EPQ suppresses H_2_O_2_ generation induced by acetaldehyde. The results indicated that H_2_O_2_ generation by the acetaldehyde increased significantly compared to that of the control (Figure 3C). However, EPQ treatment inhibited acetaldehyde-induced H_2_O_2_ production (Figure 3C). Next, we performed flow cytometry analysis to quantify the fluorescence of 2′,7′-dichloro-dihydro-fluorescein diacetate (DCFH-DA). Cellular ROS converts DCFH-DA into 2′-7′dichlorofluorescein, which has green fluorescence. PC12 cells showed significant ROS generation upon treatment with acetaldehyde, which reduced upon co-treatment with EPQ (Figure 3D). We analyzed whether EPQ affects the mitochondrial membrane potential (ΔΨm) monitored using the JC-1 dye. JC-1, an indicator dye for ΔΨm, aggregates in the mitochondria and displays red fluorescence at high ΔΨm. However, when the ΔΨm decreases, JC-1 dissociates to the monomer state at the outer membrane, resulting in green fluorescence. Acetaldehyde significantly reduced the ΔΨm, whereas EPQ treatment restored the reduction in ΔΨm (Figure 3E). EPQ treatment consistently decreased acetaldehyde-induced ROS generation in PC12 cells. These data suggest that EPQ can protect cells from oxidative stress.

### 3.3. EPQ Enhances Antioxidant Enzyme Expression in Acetaldehyde-Treated PC12 Cells

**Figure 4:**
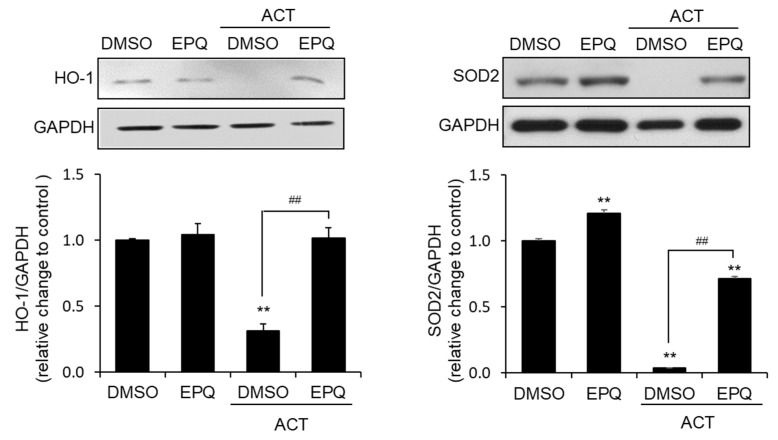
Effect of EPQ on acetaldehyde-induced oxidative stress-related enzymes in PC12 cells. Cells were treated with DMSO (0.5%, *v*/*v*) or EPQ (20 μM) in the presence and absence of acetaldehyde (500 μM) for 24 h. The oxidative stress-related protein levels of HO-1 and SOD2 were determined by western blots. GAPDH was a loading control. All values are expressed as mean ± SEM (*n* = 3; ** *p* < 0.01 compared to the DMSO control group and ^##^ *p* < 0.01 compared to the group treated only with ACT). EPQ, *ent*-peniciherqueinone; DMSO, dimethyl sulfoxide; GAPDH, glyceraldehyde 3-phosphate dehydrogenase; HO-1, heme oxygenase-1; SOD2, superoxide dismutase 2.

To confirm that the protective effect of EPQ against acetaldehyde-induced oxidative stress is related to the induction of antioxidant-related enzymes, we determined the protein levels of heme oxygenase-1 (HO-1) and superoxide dismutase 2 (SOD2). Western blot analysis confirmed that EPQ significantly increased the levels of HO-1 and SOD2 proteins, which were reduced in response to by acetaldehyde (Figure 4). These results suggest that EPQ significantly increased HO-1 and SOD2 expression in cells exposed to acetaldehyde.

### 3.4. EPQ Enhances ALDH Expression in Acetaldehyde-Treated PC12 Cells

**Figure 5:**
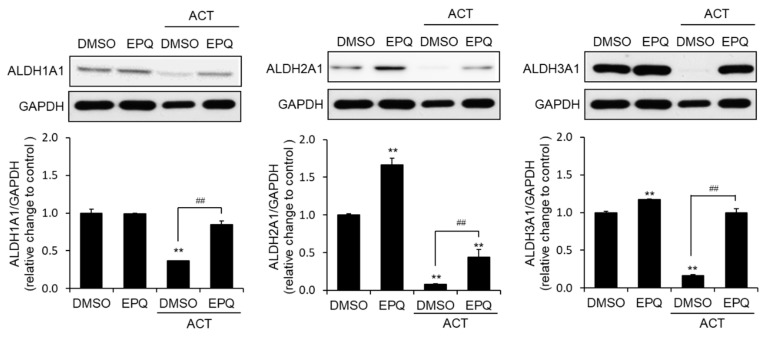
Effect of EPQ on the expression of ALDH-related proteins in acetaldehyde-treated PC12 cells. Cells were treated with DMSO (0.5%, *v*/*v*) or EPQ (20 μM) in the presence and absence of acetaldehyde (500 μM) for 24 h. The oxidative stress-related protein levels of ALDH1A1, ALDH2A1, and ALDH3A1 were determined by the western blots. GAPDH was a loading control. All values are expressed as mean ± SEM (*n* = 3; ** *p* < 0.01 compared to the DMSO control group and ^##^ *p* < 0.01 compared to the group treated only with ACT). EPQ, *ent*-peniciherqueinone; DMSO, dimethyl sulfoxide; GAPDH, glyceraldehyde 3-phosphate dehydrogenase; ALDH, aldehyde dehydrogenase.

Additionally, to confirm whether the protective effect of EPQ on acetaldehyde-induced cytotoxicity is associated with the induction of aldehyde dehydrogenase (ALDH), we analyzed the protein expression of ALDH1A1, ALDH2A1, and ALDH3A1. The western blot analysis confirmed that co-treatment of EPQ with acetaldehyde significantly increased ALDH1, ALDH2, and ALDH3 protein expression compared with cells treated with acetaldehyde alone (Figure 5). These results suggested that EPQ significantly increased ALDH1A1, ALDH2A1, and ALDH3A1 at the protein level.

### 3.5. EPQ Treatment Reduces Acetaldehyde-Induced MAPK Phosphorylation in PC12 Cells

**Figure 6:**
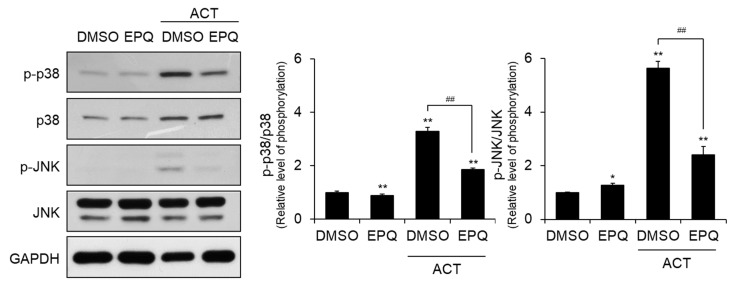
Inhibitory effect of EPQ on acetaldehyde-induced MAPK activation in PC12 cells. PC12 cells were treated with DMSO (0.5%, *v*/*v*) or EPQ (20 μM) in the presence and absence of acetaldehyde (500 µM) for 24 h. The MAPK activation-related protein levels of the phosphorylated and non-phosphorylated forms of p38 MAPK and JNK were determined in the western blots. GAPDH was a loading control. All values are expressed as mean ± SEM (*n* = 3; * *p* < 0.05, ** *p* < 0.01 compared to the DMSO control group and ^##^ *p* < 0.01 compared to the group treated only with ACT). EPQ, *ent*-peniciherqueinone; DMSO, dimethyl sulfoxide; GAPDH, glyceraldehyde 3-phosphate dehydrogenase; p38, p38 mitogen-activated protein kinase; JNK, c-Jun N-terminal kinase; p, phosphorylated.

The MAPK cascade plays important roles in oxidative stress via acetaldehyde. Accordingly, we evaluated the levels of phosphorylated (p) MAPKs in the presence and absence of acetaldehyde and EPQ. The data showed that the phosphorylated level of p38 and JNK were induced in PC12 cells upon treatment with acetaldehyde alone (Figure 6). In contrast, co-treatment with EPQ and acetaldehyde significantly reduced p38 and JNK phosphorylation. These results suggested that the inhibition of MAPK signaling by EPQ protects PC12 cells against acetaldehyde-induced cytotoxicity.

## 4. Discussion

Excessive alcohol absorption causes organ (liver, gastrointestinal tract, gonads, immune system, and brain) damage due to the formation of acetaldehyde, a metabolic product of oxidation by alcohol dehydrogenases [2,7,8,10]. Long-term accumulation of acetaldehyde exhibits serious effects on brain tissue and causes neurotoxicity and accelerates the development of neurodegenerative diseases [7,11,12]. The reasons for acetaldehyde toxicity include oxidative stress attributed to ROS generation by acetaldehyde and subsequent cell death by depletion of the oxidative defense of cells [12]. Prolonged exposure to ROS results in the dysfunction of the cell membrane system, disruption of mitochondrial potential, and eventually cell death in brain tissues [11,12]. Mitochondria present in eukaryotic cells are major components of eukaryotic cells involved in cellular respiration and are essential for the defense against oxidative stress [35]. Therefore, it may be effective to eliminate the ROS generated by acetaldehyde to prevent cell death. In the present study, it was found that acetaldehyde exposure induces ROS generation and oxidative stress. However, EPQ significantly inhibited acetaldehyde-induced cytotoxicity and ROS generation through its potent antioxidant activity in PC12 cells. Antioxidant-related enzymes, such as HO-1 and SOD2, have been reported to play an effective role in counteracting oxidative stress. EPQ also induced HO-1, SOD2, and ALDH protein levels to suppress aldehyde-induced cytotoxicity.

MAPK are serine-threonine protein kinases that play an important role in signaling from the cytoplasm to the nucleus [16,17,18,19]. MAPK signaling is regulated in a variety of cellular functions such as differentiation, proliferation, development, inflammatory response, apoptosis, and oxidative stress [25]. Typically, multiple extracellular and intracellular stimuli that simultaneously induce ROS production can activate MAPK pathways. For example, direct exposure to ROS-generation agents, such as hydrogen peroxide, ethanol, or acetaldehyde, increases the phosphorylation of MAPK pathways in several cell types [13,14]. Thus, we evaluated the phosphorylation of MAPK family proteins, including p38 MAPK and JNK [13,18,19]. Western blot analysis showed that acetaldehyde significantly activated the phosphorylation of MAPK (Figure 6). However, co-treatment of EPQ with acetaldehyde significantly inhibited the level of p-p38 and p-JNK. Based on these results, we conclude that the protective effects of EPQ include the inhibition of phosphorylated levels of MAPKs.

## 5. Conclusions

EPQ suppressed acetaldehyde-induced cytotoxicity and oxidative stress in PC12 cells by the upregulation of antioxidant-related enzymes and ALDH proteins such as HO-1, SOD2, ALDH1A1, ALDH2A1, and ALDH3A1. In addition, EPQ reduced MAPK signaling, especially p38 and JNK in acetaldehyde-treated cells. This study indicates that EPQ may serve as an efficient agent for inhibiting acetaldehyde-induced cell cytotoxicity.
